# Pulmonary Function Testing in Work-Related Asthma: An Overview from Spirometry to Specific Inhalation Challenge

**DOI:** 10.3390/ijerph18052325

**Published:** 2021-02-26

**Authors:** Mathias Poussel, Isabelle Thaon, Emmanuelle Penven, Angelica I. Tiotiu

**Affiliations:** 1 University Centre of Sports Medicine and Adapted Physical Activity, Department of Pulmonary Function Testing and Exercise Physiology, University Hospital of Nancy, University of Lorraine, F-54000 Nancy, France; 2 Development, Adaptation and Disadvantage, Cardiorespiratory Regulations and Motor Control, Department of Physiology, University of Lorraine, F-54000 Nancy, France; a.tiotiu@chru-nancy.fr; 3 Occupational Diseases Department, University Hospital of Nancy, University of Lorraine, F-54000 Nancy, France; i.thaon@chru-nancy.fr (I.T.); e.penven@chru-nancy.fr (E.P.); 4 Department of Pulmonology, University Hospital of Nancy, University of Lorraine, F-54000 Nancy, France

**Keywords:** occupational asthma, pulmonary function testing, spirometry, bronchial challenge, specific inhalation challenge

## Abstract

Work-related asthma (WRA) is a very frequent condition in the occupational setting, and refers either to asthma induced (occupational asthma, OA) or worsened (work-exacerbated asthma, WEA) by exposure to allergens (or other sensitizing agents) or to irritant agents at work. Diagnosis of WRA is frequently missed and should take into account clinical features and objective evaluation of lung function. The aim of this overview on pulmonary function testing in the field of WRA is to summarize the different available tests that should be considered in order to accurately diagnose WRA. When WRA is suspected, initial assessment should be carried out with spirometry and bronchodilator responsiveness testing coupled with first-step bronchial provocation testing to assess non-specific bronchial hyper-responsiveness (NSBHR). Further investigations should then refer to specialists with specific functional respiratory tests aiming to consolidate WRA diagnosis and helping to differentiate OA from WEA. Serial peak expiratory flow (PEF) with calculation of the occupation asthma system (OASYS) score as well as serial NSBHR challenge during the working period compared to the off work period are highly informative in the management of WRA. Finally, specific inhalation challenge (SIC) is considered as the reference standard and represents the best way to confirm the specific cause of WRA. Overall, clinicians should be aware that all pulmonary function tests should be standardized in accordance with current guidelines.

## 1. Introduction

Asthma is defined as a heterogeneous disease, usually characterized by chronic airway inflammation [1]. This includes a history of respiratory symptoms (recurrent episodes of wheezing, shortness of breath, chest tightness, and cough) that vary in intensity and over time, associated with variable expiratory airflow limitation. Numerous different underlying disease processes have for now been identified in asthma, leading to the description of “asthma phenotypes” according to clinical and pathophysiological characteristics. In the occupational setting, the convergence of notable advances in respiratory pathophysiology and in the field of immunology has allowed the identification of a specific condition termed work-related asthma (WRA). This common occupational lung disease refers to asthma specifically caused by an inciting exposure in the workplace (occupational asthma, OA) but also to asthma that is worsened by workplace conditions (work-exacerbated asthma, WEA) [2]. Overall, as for asthma, WRA has to be rigorously investigated from diagnosis to follow up. Detailed clinical history and precise work exposure history are required to suggest the relationship between asthma and exposures in the work setting and to identify likely exposure(s) able to cause WRA. Clinical features alone, however, are insufficient to accurately diagnose WRA, and should be associated with more objective investigations such as allergy testing (such as assessment of serum allergens-specific IgE antibodies or skin prick tests) and exploration regarding underlying airway inflammation (such as fractional exhaled nitric oxide (FeNO) or sputum eosinophil count). Finally, lung function testing to document variable expiratory airflow limitation undoubtedly represents a key step of the diagnosis of WRA [1,2].

From the simple vital capacity measurement to the first published description of forced expiratory maneuver, lung function testing has become increasingly integrated in the mainstream of clinical medicine [3,4,5]. Technological advances and computerization have also brought great benefits in term of accuracy and convenience in pulmonary function testing [6]. If the pattern of respiratory symptoms is important, the clinical history alone is not sufficient to diagnose WRA. Lung function testing should be carried out to objectively assess the presence of asthma, the possible association between asthma and workplace exposure, and the suspected specific causal agent involved [1,2,7]. Spirometry, tests for reversibility such as methacholine, or histamine inhalation challenge tests widely help to confirm asthma but may also be normal in WRA and could not be used to exclude the diagnosis [8]. In order to identify the workplace as the cause of the respiratory symptoms, serial peak flow measurements on days at and away from work should be considered as recommended in guidelines [8,9]. Changes in non-specific bronchial hyper-responsiveness (NSBHR) at and away from work may also be informative but with a moderate sensitivity and specificity for diagnosis [10,11,12]. Finally, specific inhalation challenge (SIC) is considered as the reference standard for the diagnosis of sensitizer-induced occupational asthma in patients with a history of work-related symptoms and represents the best way to confirm the specific cause of WRA [13]. All these respiratory function tests may help clinicians to objectively assess variable expiratory airflow limitation and accurately diagnose WRA (Table 1).

As pulmonary function testing is a key tool in the diagnosis of WRA, we aimed to give an overview of the different and graded tests available that should be considered in order to accurately diagnose WRA. Systematic reviews, meta-analyses, and trials dealing with lung function testing and WRA were searched in PubMed and Cochrane Library databases by using the medical subject headings (MeSH) terms “Respiratory Function Tests” and “Asthma, Occupational” from January 2010 to September 2020. The selection of articles was based on authors’ expertise and additional relevant studies in the literature known to the authors were also included for this overview.

## 2. Initial Lung Function Assessments When WRA Is Suspected

Spirometry, bronchodilator responsiveness testing, and bronchial provocation testing to assess NSBHR generally represent the first line clinical tests of respiratory function aiming to confirm asthma when WRA is suspected. In addition to the analysis of clinical features of patients and to a review of the workplace environment, further pulmonary function testing should then be decided in order to highlight the possible association between asthma and work exposure [8,14].

### 2.1. Spirometry and Bronchodilator Responsiveness Testing

Every worker with suspected WRA should perform spirometry according to the best guidelines to identify variable airflow limitation [15,16]. In addition to these available and general guidelines (i.e., providing objective measurements in the diagnosis of respiratory diseases and in the assessment of lung health), special considerations relevant to the quality and interpretation of spirometry should be considered in the field of the work setting, now considered in the 2019 updated standards [14,15]. Before any measurements, the laboratory environment should be carefully recorded (i.e., ambient temperature and barometric pressure) and spirometers have to meet the standards of ISO 26782. Device quality assurance and calibration (for both flow and volume-based sensors) should be checked daily and testing should be performed by a trained and experienced operator. Before testing, patients should avoid some activities as well as withhold some specific medications [14,15,16]. Patients’ details (age, height, weight, and ethnicity) should be recorded for determination of calculated predicted values. Before and during tests, patients should be as comfortable as possible. The test procedure includes a forced vital capacity (FVC) maneuver usually consisting of the succession of three phases: (1) a maximal inspiration that should be rapid and with the shortest pause at full inspiration as possible (≤2 s), followed by (2) an “explosive” full and continuous expiration until a plateau has been reached (or forced expiratory time reaches 15 s), and finally, (3) a full inspiration at maximal flow. Acceptability, usability, and repeatability criteria for FVC and forced expiratory volume in the first second (FEV_1_) should be satisfied allowing three acceptable FEV_1_ and FVC measurements with a maximum of eight consecutive maneuvers [8,14,15].

Current FEV_1_, FVC, and FEV_1_/FVC values measured should be compared to predicted values and the lower limit of normal (LLN) with the accepted definition of a reduced FEV_1_/FVC ratio below the 5th percentile of the predicted value for an obstructive ventilatory defect [17]. At the time of initial evaluation, spirometry could be either normal or showing expiratory airflow limitation. In this latter case, bronchodilator responsiveness testing should be performed in order to evaluate the degree of improvement of airflow with attention paid to regular treatment of patients (especially bronchodilators that should be withheld). Bronchodilator administration generally consists of using short-acting b2-agonists such as salbutamol (four successive doses of 100 µg). Three or more additional acceptable post-bronchodilator spirometry tests should then be repeated after a delay of 15 min. A positive bronchodilator response is based on the increase in FEV_1_ and/or FVC ≥ 12% from baseline and ≥200 mL [15,16,17].

Overall, and in the work setting, the presence of expiratory airflow limitation associated with a positive bronchodilator response is consistent with the diagnosis of asthma. However, normal spirometry could not rule out the diagnosis of asthma and if no variable airflow limitation is shown at initial assessment, further bronchial assessments should be performed to identify the presence of NSBHR [2,7,8].

### 2.2. Nonspecific Bronchial Hyper-Responsiveness (NSBHR)

Bronchial provocation testing to assess airway hyper-responsiveness (defined as an exaggerated response to stimuli that cause airway narrowing) is a valuable tool in the evaluation of WRA [7,18]. Bronchial challenge tests can be performed by using either direct or indirect agents [19]. Methacholine (acetyl-ß-methylcholine chloride) is generally used as the agent of choice for the direct challenge test (but histamine can also be used), as it mimics the physiological neurotransmitter acetylcholine on airway smooth muscle receptors, leading to airway narrowing. Compared to individuals with normal airway responsiveness, workers with NSBHR show airway narrowing for lower inhaled dose of methacholine and also with a marked degree. Bronchial challenge testing can also be performed using indirect agents (such as mannitol) but less data are available regarding those latter challenges [20]. For instance, mannitol bronchial challenge testing has been shown to be useful to detect asymptomatic patients in the workplace but with hyper-responsiveness that could be at risk of developing WRA [21]. Overall, bronchial challenge testing has to meet technical standards with rigorous methodology, with respect to accepted indications and contraindications [19,22]. Due to its high negative predictive value, methacholine challenge testing mostly helps to exclude the diagnosis of asthma. Regarding the fall of FEV_1_ for an increasing dose (PD_20_; provocative dose causing a 20% fall in FEV_1_) or concentration (PC_20_; provocative concentration causing a 20% fall in FEV_1_) of methacholine, NSBHR is categorized from normal (PD_20_ > 2 μmol; PC_20_ > 16 mg/mL) to marked (PD_20_ < 0.03 μmol; PC_20_ < 0.25 mg/mL) [19]. In the field of suspected WRA, assessment for NSBHR should initially be performed while the patient is still working (i.e., still exposed to the suspected offending agent(s)) because NSBHR may rapidly return to normal once exposure ceases, and recur after the re-exposure. A basal positive methacholine challenge supports the diagnosis of WRA in patients with asthma symptoms. However, the absence of NSBHR does not exclude a diagnosis of OA, particularly when the subjects are tested after they have been away from work [23]. Compared to SIC, single NSBHR tests have a relatively high sensitivity (84%) and negative predictive value (75%) but a low specificity (48%) in predicting the diagnosis of OA [18]. The sensitivity and the negative predictive value of the NSBHR test are significantly increased when assessed at least once while people are still at work [24]. Finally, adding induced sputum and exhaled nitric oxide measures as well as the NSBHR test increased the OA diagnostic sensitivity to 94% compared to a sensitivity of 87% with the NSBHR test alone [25].

## 3. Specific Functional Respiratory Tests to Consolidate WRA Diagnosis and to Differentiate OA from WEA

### 3.1. Serial Peak Expiratory Flow (PEF)

One of the key procedures during spirometry is the “explosive”, full and maximal expiration that allows a valid peak expiratory flow (PEF). Indeed, PEF should be achieved with a rise time <150 ms to meet accepted guidelines, therefore requiring continuous encouragement to fully and sharply expire [15,16]. Under such conditions, recording PEF during working and non-working periods can be helpful to highlight work-related changes and therefore, add further evidence for an association between asthma and work exposure [9,26]. Even if serial PEF records are not universally accepted (mostly because of moderate reliability), evaluation by experienced respiratory and/or occupational physicians coupled with computer assistance analysis in changes in PEF values is widely admitted as a diagnostic aid for WRA [9,27,28]. Serial PEF measurements should then be performed over a continuous 4- to 6-week period (including at least a 2-week non-working period) with four PEF records a day. Computer assistance for analyzing PEF records such as the occupation asthma system (OASYS)-2 allows a sensitivity of 75% and a specificity of 94% in the diagnosis of WRA [26,29,30]. Furthermore, indexes and interpretation of OASYS scores may help in distinguishing OA from WEA with a score >2.5 in favor of OA, a score ranging from 1.5 to 2.5 in favor of WEA, and a score ≤1.4 not in favor of WEA. In a systematic review for serial PEF measurements in the diagnosis of OA, a sensitivity of 82% and a specificity of 88% have been shown [31]. The addition of sputum cell counts to PEF monitoring has also been shown to be useful to improve the diagnosis of OA [32]. Based on serial PEF measurements, another diagnosis score for OA has also been proposed. Based on computed PEF analysis on days on and out of work, calculation from the area between the curves (ABC) of PEF allows the elaboration of a score (ABC score) that can be useful in the diagnosis of OA [33,34]. A score of 15 L/min/h between rest and workdays allows the highest specificity and a score of 5.6 L/min/h provides a good combination of reasonable sensitivity and specificity. As the calculation of the ABC score needs shorter PEF surveillance (compared to the OASYS score) [35], it should also improve compliance among workers with suspected OA. On the other hand, PEF has also been shown to have some limitations, especially regarding to poor reliability [28,36]. Overall, serial PEF measurements are generally considered a useful and objective confirmatory test for OA that can be achieved by two-thirds of patients asked to perform them. In light of the available literature, serial PEF measurements appear to be feasible, sensitive, and specific in the occupational setting.

### 3.2. Serial NSBHR Challenge

Another way to highlight the possible link between asthma and work exposure is to perform serial bronchial challenges during periods at and off work [2,37,38]. Respecting the standards for bronchial challenge testing, comparison of NSBHR between a challenge performed while the patient is currently exposed at work and after a 10- to 14-day period away from any work exposure may be informative in the diagnosis of WRA [10,19,22,32]. Even if this approach of serial NSBHR has shown moderate sensitivity (43–62%) and specificity (52–83%), it is generally accepted that a three-fold increase in the PD_20_ or PC_20_ for a positive test performed out of work (compared to baseline values performed during a period of work exposure) is considered significant and consistent with a diagnosis of OA [2,37,38,39].

### 3.3. Specific Inhalation Challenge (SIC)

Initially developed in the early 1970s by Pepys et al., SIC testing consists of the controlled exposure of a patient to an agent suspected of sensitizer-induced (i.e., immunologically mediated) OA [40,41]. SIC should be performed under laboratory conditions and meet standards for methodology, assessment of bronchial response, and interpretation of SIC results [13]. SIC should only be performed in hospital-based specialized centers because it requires a high level of safety. Indeed, in case of excessive reactions (immediate and late phases), appropriate and readily available management should be offered to patients, therefore needing trained personnel and experienced physician supervision. As for spirometry and non-specific bronchial provocation testing, specific medications able to attenuate/inhibit bronchial response should be withdrawn prior to SIC [13,42]. Ideally, SIC should be performed within 4 successive days as follows: first, a “control” day exposing the patient to a control substance, followed by 2 consecutive days aimed to deliver the suspected occupational agent in the nearest conditions to the workplace exposure, and finally, a last day for assessing post-SIC NSBHR. The “control” day has two important goals: to check functional stability, which is absolutely necessary for the correct interpretation of changes in FEV_1_, and also to provide a comparison point for any reactions to the tested active agent. Delivery of the occupational agent can be conducted by different ways, depending on the nature of the agent tested, but always as close as possible to the workplace conditions [43,44]. More information on the techniques used for SIC with different occupational agents such as the form and nature of the active and control agents and on methods, quantities, and duration of delivery can be found in the handbook of procedures for SIC testing, available as supplementary material, of the European Respiratory Society Task Force [13,45]. During SIC, the concentration of the occupational agent should not exceed known occupational exposure limits, and duration of exposure should increase very gradually from 10 s to 60 min (i.e., 10 s, 1 min, 5 min, 10 min, 15 min, 30 min and 60 min). Following each duration of exposure, bronchial response should be assessed with measurement of FEV_1_. If the decrease in FEV_1_ is 10% compared to baseline, FEV_1_ should be re-assessed 10 to 20 min after, before continuing the test. At the end of the challenge, FEV_1_ should be assessed at 10–15 min intervals during the first hour, then every 30–60 min for the next following 6 h. It is generally admitted that an SIC is considered positive when bronchial assessment shows a sustained decrease in FEV_1_ of at least 15% from the pre-challenge value. If SIC shows no “significant” fall in FEV_1_ (i.e., fall < 15%), NSBHR challenge can be performed on the last day and a two- to three-fold decrease in the PD_20_ or PC_20_ compared to baseline values can be suggestive of a positive SIC [13,38]. Even though SIC is the most expensive technique for the diagnosis of OA, it is considered as the reference standard with an assumed 100% accuracy [46]. The European Respiratory Society Task Force recommends performing SIC with an occupational agent for confirmation of the diagnosis of OA when other objective methods are not feasible, less efficient, or have failed to prove the diagnosis of OA [13].

## 4. Conclusions

Due to relevant and considerable advances in pulmonary function testing during the last few decades, clinical tests of respiratory function have become increasingly integrated in the mainstream of clinical medicine. In addition to the clinical presentation and the pattern of respiratory symptoms, pulmonary function tests represent valuable tools in the field of WRA for assessing the presence of asthma, the possible association between asthma and the workplace, and the suspected specific causal agent involved. Physicians involved in the management of WRA should be aware of the wide spectrum of available tests, from spirometry to SIC. Clinicians should, therefore, know the accepted standards for the completion and interpretation of all these respiratory function tests, which may help them to objectively assess variable expiratory airflow limitation and accurately diagnose WRA (Table 1).

## Figures and Tables

**Table 1:** Pulmonary function testing for the evaluation of work-related asthma.

First Line (Initial) Evaluation
Spirometry
Bronchodilatator Responsiveness Testing
Non-specific Bronchial Hyper-Responsiveness (NSBHR) challenge
**Second Line (Specific) Evaluation**
Serial Peak Expiratory Flow (PEF)
Serial NSBHR challenge
Specific Inhalation Challenge (SIC)

## Data Availability

Not applicable.
